# Isolation and Identification of Compounds from Bioactive Extracts of* Taraxacum officinale* Weber ex F. H. Wigg. (Dandelion) as a Potential Source of Antibacterial Agents

**DOI:** 10.1155/2018/2706417

**Published:** 2018-01-01

**Authors:** Katy Díaz, Luis Espinoza, Alejandro Madrid, Leonardo Pizarro, Rolando Chamy

**Affiliations:** ^1^Departamento de Química, Universidad Técnica Federico Santa María, Av. España No. 1680, Valparaíso, Chile; ^2^Departamento de Química, Facultad de Ciencias Naturales y Exactas, Universidad de Playa Ancha, Avda. Leopoldo Carvallo 270, Playa Ancha, Valparaíso, Chile; ^3^Facultad de Ingeniería, Escuela de Ingenieria Bioquímica, Pontificia Universidad Católica de Valparaíso, General Cruz 34, Valparaíso, Chile; ^4^Fraunhofer Chile Research Foundation-Centre for Systems Biotechnology (FCR-CSB), Mariano Sánchez Fontecilla 310, Las Condes, Santiago, Chile

## Abstract

Currently, the most effective treatment for recurrent urinary tract infections in women is antibiotics. However, the limitation for this treatment is the duration and dosage of antibiotics and the resistance that bacteria develop after a long period of administration. With the aim of identifying mainly novel natural agents with antibacterial activity, the present study was undertaken to investigate the biological and phytochemical properties of extracts from the leaves* Taraxacum officinale.* The structural identification of compounds present in hexane (Hex) and ethyl acetate (AcOEt) extracts was performed by mass spectrometry (GC-MS) spectroscopic techniques and nuclear magnetic resonance (NMR) with the major compounds corresponding to different sesquiterpene lactones (*α*-santonin, glabellin, arborescin, and estafiatin), monoterpene (9,10-dimethyltricycle [4.2.1.1 (2,5)]decane-9,10-diol), phytosterol (Stigmasta-5,22-dien-3*β*-ol acetate), terpenes (lupeol acetate, pregn-5-en-20-one-3*β*-acetyloxy-17-hydroxy, 2-hydroxy-4-methoxy benzaldehyde), and coumarin (benzofuranone 5,6,7,7-a-tetraaldehyde-4,4,7a-trimethyl). The results obtained show that the Hex extract was highly active against* Staphylococcus aureus* showing a MIC of 200 *μ*g/mL and moderately active against* Escherichia coli *and* Klebsiella pneumoniae* with MIC values of 400 *μ*g/mL and 800 *μ*g/mL for the other Gram-negative strains tested with* Proteus mirabilis *as uropathogens* in vitro*. Therefore, the effective dandelion extracts could be used in the development of future products with industrial application.

## 1. Introduction

The increasing resistance of uropathogens to antibiotics and the recognition of the generally self-limiting nature of uncomplicated urinary tract infection (UTI) suggest that it is time to reconsider the empirical treatment of UTI by using antibiotics. Limitation for this treatment is the duration and dosage of antibiotics and the resistance that bacteria develop after a long period of administration [1]. Therefore, alternatives to the pharmaceutical industry approaches need to be considered. During our continuous exploratory search for new antibacterial extracts, we have selected the Chilean medicinal plant* Taraxacum officinale* Weber ex F. H. Wigg. (Asteraceae), commonly known as dandelion, for a systematic study on the chemical composition and potential antibacterial properties.

*T. officinale* is an herbaceous perennial plant of the family Asteraceae, native to Asia; it can be found growing in temperate regions of the world, including Chile in the III-IX region. Dandelion is considered a weedy species [2]; it has numerous antioxidant, anti-inflammatory, antidiabetic, antimicrobial, and anticancer properties [3, 4]. There are several references to treating bacterial infections and the use of traditional botanical remedies [5–7]. These properties have been attributed to the large number of bioactive compounds in their tissues, and several studies have reported a wide range of compounds, including terpenes, flavonoids, and phenolic compounds, which are mentioned as responsible for the medicinal activity of different plants [8, 9]. For the genus* Taraxacum*, only few studies concerning its antimicrobial properties consider chemical identification of the extracts obtained and, most of the time, this identification is qualitative (e.g., using colorimetric methods indicating presence or absence). Authors report the presence of terpenoids, triterpenoids, steroids, coumarins, phenols, saponins, flavonoids, flavones, flavonols, chalcones, phlobatannins, and cardiac glycosides in antimicrobial extracts [10–18], but neither compound isolation nor further identification was performed.

Therefore, based on the above reasoning and observations, the aim of the study was to explore the phytochemical of n-hexane and ethyl acetate extracts of the* Taraxacum officinale *plant and its antibacterial activity against selected uropathogenic bacterial strains.

## 2. Results and Discussion

### 2.1. Chromatographic Analysis

Results of the gas chromatography analysis of hexane and ethyl acetate extracts from leaves of* T. officinale* are summarized in Tables 1 and 2, respectively.

Forty components were identified in the hexane extract: 72.46% were triterpenoids, 16.56% terpenes, 4.25% phthalate ester, 1.46% fatty acids and derivatives, 1.42% aldehydes and ketones, 1.81% alcohols, and 0.55% unknown compounds. The hexane extract was mainly characterized by lupeol acetate (25.09%), betulin (23.81%), lupeol (23.31%), 3,7,11,15-tetramethyl-2-hexadecen-1-ol (13.08%), diethyl phthalate (4.25%), and phytol (3.48%). On the other hand, eighty components were identified in the ethyl acetate extract: 37.06% were triterpenoids, 17.08% terpenes, 3.04% fatty acids and derivatives, 1.50% ketones and alcohols, and 37.86% unknown compounds. The ethyl acetate extract was mainly characterized by lupeol acetate (19.95%), 3,7,11,15-tetramethyl-2-hexadecen-1-ol (15.57%), betulin (6.17%), *α*-Amyrin (4.78%), *β*-sitosterol (4.55%), and (22Z)-Stigmasta-5,22-dien-3*β*-ol acetate (2.98%). In addition, the composition of both extracts of leaves from* T. officinale* in which triterpenoids were the predominant portion found was reported, unlike other studies in which it has been described that most compounds constituting the leaf part are polyphenols and flavonoids glycosides [4].

**Figure 1:**
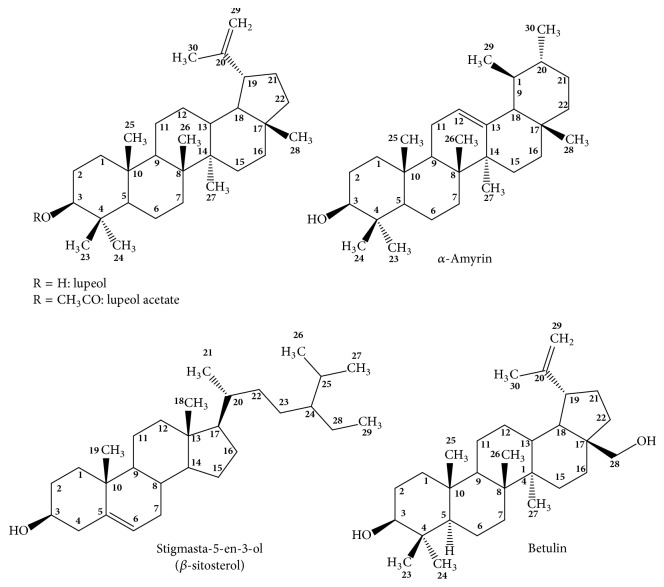
Structures of main terpenes isolates of n-hexane and ethyl acetate extracts.

Most of the metabolites isolated belonging to the group of terpenes (lupeol, lupeol acetate, *α*-Amyrin, *β*-sitosterol, and betulin) from both extracts were characterized and identified by different NMR experiments (^1^H-NMR, ^13^C NMR 1D, and 2D). In addition, all the spectra of these products were compared with literature data and with authentic samples acquired from Sigma-Aldrich. The structures of these terpenes are shown in Figure 1.

### 2.2. Spectroscopic NMR Characterization

#### 2.2.1. Lupeol

^1^ *H-NMR*. 4.68 (1H, bs, H-29a); 4.56 (1H, bs, H-29b); 3.18 (1H, t, H-3); 2.36 (1H, m, H-19); 1.67 (3H, s, H-30); 1.62 (2H, m, H-2); 1.38 (2H, m, H-6); 1.35 (1H, dd, H-18); 1.25 (1H, t, H-9); 1.02 (3H, s, H-26); 0.96 (3H, s, H-23); 0.94 (3H, s, H-27); 0.82 (3H, s, H-25); 0.78 (3H, s, H-28); 0.67 (1H, t, H-5).

^13^ *C-NMR*. 151.2 (C-20); 109.5 (C-29); 79.2 (C-3); 55.5 (C-5); 50.6 (C-9); 48.5 (C-18); 48.2 (C-19); 43.2 (C-17); 43.0 (C-14); 41.0 (C-8); 40.2 (C-22); 39.0 (C-4); 38.9 (C-1); 38.2 (C-13); 37.3 (C-10); 35.8 (C-16); 34.4 (C-7); 30.0 (C-21); 28.2 (C-23); 27.2 (C-15); 27.6 (C-2); 25.3 (C-12); 21.1 (C-11); 19.5 (C-30); 18.5 (C-6); 18.1 (C-28); 16.3 (C-26); 16.2 (C-25); 15.6 (C-24); 14.7 (C-27) [19–22].

#### 2.2.2. Lupeol Acetate

^1^ *H-NMR*. 4.69 (1H, d,* J* = 2.1 Hz, H-29a); 4.57 (1H, m, H-29b); 4.48 (1H, dd, *J* = 10.5 and 6.6 Hz, H-3); 2.37 (1H, dt, *J* = 11.1 and 5.7 Hz, H-19); 2.04 (3H, s, CH_3_CO); 1.68 (3H, s, H-30); 1.03 (3H, s, H-26); 0.94 (3H, s, H-27); 0.88 (3H, s, H-24); 0.88 (3H, s, H-25); 0.70 (3H, s, H-28).

^13^ *C-NMR*. 171.4 (CO); 151.4 (C-20); 109.7 (C-29); 81.3 (C-3); 55.7 (C-5); 50.7 (C-9); 48.3 (C-18); 48.3 (C-19); 43.2 (C-17); 42.8 (C-14); 41.2 (C-8); 40.3 (C-22); 38.7 (C-1); 38.4 (C-4); 38.1 (C-13); 37.4 (C-10); 34.5 (C-16); 34.5 (C-7); 30.2 (C-21); 28.3 (C-23); 27.8 (C-2); 25.4 (C-15); 25.4 (C-12); 21.3 (C-11); 21.1 (CH3CO); 19.6 (C-30); 18.5 (C-6); 18.3 (C-28); 16.8 (C-24); 16.5 (C-25); 16.3 (C-26); 14.8 (C-27) [23–25].

#### 2.2.3. *α*-Amyrin

^1^ *H-NMR*. 5.06 (1H, t, *J* = 3.2 Hz, H-12); 3.16 (1H, dd, *J* = 11.2 and 5.1 Hz, H-3); 1.94 (1H, dt, *J* = 13.5 and 4.5 Hz, H-15*β*); 1.85 (1H, dt, *J* = 7.0 and 3.0 Hz, H-22); 1.76 (1H, dt, *J* = 13.5 and 5.0 Hz, H-16*β*); 1.01 (3H, s, H-27); 0.94 (3H, s, H-28); 0.93 (3H, s, H-23); 0.89 (3H, s, H-26); 0.85 (3H, d, *J* = 6.0 Hz, H-29); 0.73 (3H, d, *J* = 7.0 Hz, H-30); 0.67 (1H, d, *J* = 11.6 Hz, H-5).

^13^ *C-NMR*. 139.5 (C-13); 124.4 (C-12); 79.6 (C-3); 59.0 (C-18); 55.1 (C-5); 47.7 (C-9); 42.0 (C-14); 41.5 (C-22); 40.0 (C-8); 39.6 (C-19); 39.6 (C-20); 38.7 (C-1); 38.7 (C-4); 36.9 (C-10); 33.7 (C-17); 32.2 (C-7); 31.2 (C-21); 28.7 (C-2); 28.1 (C-28); 28.1 (C-23); 27.2 (C-15); 26.6 (C-16); 23.3 (C-11); 23.2 (C-27); 21.4 (C-30); 18.4 (C-6); 17.4 (C-29); 16.8 (C-26); 15.6 (C-25); 15.6 (C-24) [20, 22, 26, 27].

#### 2.2.4. Stigmasta-5-en-3-ol (*β*-Sitosterol)

^1^ *H-NMR*. 5.36 (1H, t, *J* = 6.4 Hz, H-6); 3.53 (1H, tdd, *J* = 4.5, 4.2 and 3.8 Hz, H-3); 1.01 (3H, d, *J* = 7.2 Hz, H-29); 0.93 (3H, d, *J* = 6.5 Hz, H-19); 0.84 (3H, t, *J* = 7.2 Hz, H-24); 0.83 (3H, d, *J* = 6.4 Hz, H-26); 0.81 (3H, d, *J* = 6.4 Hz, H-27); 0.68 (3H, s, H-18).

^13^ *C-NMR*. 140.9 (C-5); 121.9 (C-6); 72.0 (C-3); 56.9 (C-14); 56.3 (C-17); 50.3 (C-9); 46.1 (C-22); 42.6 (C-13); 42.5 (C-4); 39.9 (C-12); 37.5 (C-1); 36.7 (C-10); 36.3 (C-18); 34.2 (C-20); 32.1 (C-7); 32.1 (C-8); 31.9 (C-2); 29.4 (C-25); 28.5 (C-16); 26.3 (C-15); 26.3 (C-21); 23.3 (C-23); 21.3 (C-11); 20.1 (C-26); 19.6 (C-27); 19.2 (C-19); 19.0 (C-28); 12.2 (C-24); 12.0 (C-29) [28, 29].

#### 2.2.5. Betulin

^1^ *H-NMR*. 4.70 (1H, bs, H-29b); 4.58 (1H, bs, H-29a); 3.79 (1H, d, *J* = 10.8 Hz, H-28b); 3.33 (1H, d, *J* = 10.8 Hz, H-28a); 3.18 (1H, dd, *J* = 10.2 and 5.3 Hz, H-3); 1.67 (3H, s, H-30); 0.99 (3H, s, H-27); 0.97 (3H, s, H-26); 0.96 (3H, s, H-23); 0.80 (3H, s, H-25); 0.75 (3H, s, H-24).

^13^ *C-NMR*. 150.6 (C-20); 109.8 (C-29); 79.2 (C-3); 60.6 (C-28); 55.4 (C-5); 50.5 (C-9); 48.8 (C-19); 47.9 (C-17); 47.9 (C-18); 42.8 (C-14); 41.0 (C-8); 38.9 (C-1); 38.8 (C-4); 37.4 (C-10); 37.2 (C-13); 34.3 (C-7); 34.1 (C-22); 29.8 (C-21); 29.2 (C-16); 28.1 (C-23); 27.5 (C-2); 27.1 (C-15); 25.3 (C-12); 20.9 (C-11); 19.2 (C-30); 18.4 (C-6); 16.2 (C-25); 16.1 (C-26); 15.4 (C-24); 14.8 (C-27) [30, 31].

Recently, other authors have determined from ethanol extract three novel lupane-, bauerane-, and euphane-type triterpenoids and other known triterpenes present in dandelion roots, using spectroscopic analysis, which have potential anti-inflammatory activity [32, 33]. Dandelion leaves in this study were found to be rich in triterpenes such as betulin, lupeol acetate, lupeol, *α*-Amyrin, and *β*-sitosterol from both extracts with potential antibacterial activity against pathogens that infect the urinary tract. In several investigations, the toxicity of dandelion was found to be low, due to absence of any significant toxins or alkaloids [4]. Therefore, it would be interesting to improve the biological activity by enhancing the extract with other additives to develop a pharmaceutical product for medicinal purposes.

### 2.3. Determination of the Fatty Acids

**Figure 2:**
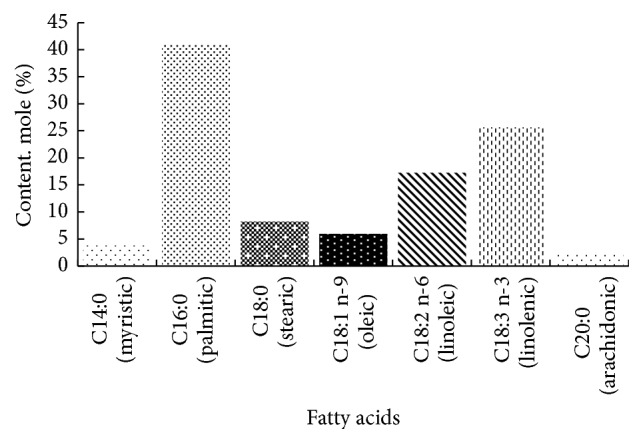
Fatty acid (% w/w of total fatty acids) composition in the leaves of* T. officinale* from Temuco region, Chile. The values represent the means of three samples, analysed individually in triplicate.

It was determined that the total fatty acid content of the dandelion leaves, using the Soxhlet method, is 4.8 ± 0.5% raw extract/dry matter, composed of different fatty acids as shown in Figure 2 and Table 3.

Figure 2 shows the fatty acid composition of leaves sampled from* T. officinale*; seven fatty acids were identified in the raw extracts, from which the most abundant was palmitic acid (16 : 0) with 41 ± 0.8%. The next most abundant fatty acids were linolenic acid (18 : 3n-3) and linoleic acid (18 : 2n-6), corresponding to 26 ± 1.9% and 17 ± 1.0%, respectively. Small and very small amounts of stearic (18 : 0), oleic (18 : 1n-9), myristic (14 : 0), and arachidic (20 : 0) acids were also detected.

Manning 2001 [34] mentioned that pollen lipids, dominated by long-chain unsaturated (linoleic and linolenic) and saturated (myristic and lauric) fatty acids, have bactericidal and antifungal properties, playing an important role in inhibiting the growth of the spore forming bacteria* Paenibacillus *(American foulbrood),* Melissococcus pluton* (European larvae foulbrood), and other microorganisms that inhabit the brood combs of beehives.

The total fatty acid content in leaves, relative to dry plant weight, is 3,9 mg per gram and palmitic acid 1.5 mg per gram as the prevailing fatty acid in the sample. The next two most abundant fatty acids were 18 : 2n-6 and 18 : 3n-3 (Table 1). These values are in accordance with those reported by Imai et al. (1995), Liu et al. (2002), and USDA (2016) [35–37].

### 2.4. *In Vitro* Antimicrobial Assay

The antibacterial effects of the extracts of* T. officinale* were evaluated against four uropathogenic bacteria (Table 4). Evaluation of the dandelion extracts revealed that the extract Hex inhibited the growth of* S. aureus* by 89% at 200 *μ*g/mL and was a more effective inhibitor for the Gram-positive than for the Gram-negative strains (Table 4).* K. pneumoniae* was the lowest inhibited by the Hex extracts.

EtOAc extract showed low activity against* E. coli.* with 1600 *μ*g/mL and growth inhibition with a 97% value. However, this extract did not exert an antibiotic effect on Gram-positive bacteria* S. aureus* in the concentrations tested (Table 4). Similar results have been reported where the ethanol extract of leaves of* T. officinale* had low antimicrobial activity against* S. aureus, E. coli,* and* Salmonella abony* [38].

In addition, it was observed that as the extract concentration increased, so does the bacteria growth inhibition. This inhibitory effect may be due to the presence of phenolic compounds, terpenes, tannins, flavonoids, alkaloids, and/or proteins in the plant extracts. Such compounds had been reported to have an active effect on the bacterial cells membrane, which may destroy these microorganisms [39, 40].

In studying the antipathogenic properties of genus* Taraxacum *to combat infectious diseases,* T. officinale* is the most studied species, but it has shown various results depending on the extraction characteristics or on the bioassay performed. For example, a methanolic extract of* T. officinale* at 0.2 mg/mL was effective as antibacterial against* Micrococcus luteus* and* Vibrio cholera *with MIC values of 1.0 mg/mL and 12.5 mg/mL, respectively, but did not show any activity against* S. aureus*,* Enterobacter faecalis*,* Enterococcus* bacteria*, V. cholera*,* Bacillus subtilis*,* Pseudomonas aeruginosa*,* K. pneumonia*, or* E. coli* [12]. In another work, methanolic extracts* of T. officinale *show an activity between 0.003 and 0.5 mg/mL on* S. aureus, P. aeruginosa, B. cereus, Shigella sonnei, S. enterica *serovar Typhimurium*, E. coli, K. pneumonia, Candida albicans,* and* C. neoformans* with MIC values ranging from 0.04 to 5.0 mg/mL [11]. So, these extracts could be active or improve their antibacterial activity against other Gram-negative and Gram-positive strains, and this work provides preliminary information for the development and use of natural medicine with extracts of* T. officinale* in the control of disease against uropathogenic bacteria, as antimicrobial agents.

## 3. Materials and Methods

### 3.1. Collection and Plant Material

The leaves of* T. officinale *were collected in Temuco, Cautín province (IX region), Chile, in May 2014 and authenticated by agronomist Lorena Jorquera, Ph.D., from “Pontificia Universidad Católica de Valparaíso”, V region, Chile. The leaves were transported to the laboratory, washed 3 times with water and once with sterile distilled water (WDS), then air-dried on kraft paper, and placed in hot air oven at a temperature of 45.0°C for a period of 4 days until the weight became constant. The dried plant material was converted to a powdered form with the help of clean grinder.

### 3.2. Preparation of the Extracts

The extracts of* T. officinale *(1,5 g, 1,5%) were obtained from dried plant material (100 g) suspended first in 400 mL of n-hexane and then the residues were further extracted with ethyl acetate (EtOAc) separately; that is, an extraction was performed sequentially and serially. The extraction was carried out by using an orbital shaker (150 rpm) at 25°C for 48 h at room temperature; then the extract was filtered through Whatman N° 1 filter paper (Sigma-Aldrich, Darmstadt, Germany), concentrated under reduced pressure with a rotatory evaporator, and finally kept in the refrigerator (−4°C) until the analyses were made.

### 3.3. Isolation of Natural Compounds from Extracts

The n-hexane and EtOAc extracts were subjected to chromatography over a silica gel column (400 g) and eluted with mixtures of increasing polarity n-hexane and EtOAc. Fractions were combined based on TLC monitoring and purified by repeated CC on silica gel columns. Compounds were identified by chromatographic analysis and spectroscopic data, including ^1^H- and ^13^C-NMR, respectively, on a Bruker Avance 400 Digital NMR spectrometer operating at 400.1 MHz for ^1^H and 100.6 MHz for ^13^C, and comparisons with data reported in the literature were made.

### 3.4. Chromatographic Analysis

The n-hexane and ethyl acetate extract was diluted with acetone, and analysis by gas chromatography (Hewlett Packard, Palo Alto, CA, USA) was carried out according to the method detailed elsewhere [41]. The operating conditions were as follows: on-column injection; injector temperature, 250°C; detector temperature, 280°C; carrier gas, He at 1.0 mL/min; oven temperature program, 40°C increased to 260°C at 4°C/min and then 260°C for 5 min, to achieve the best separation through a capillary Rtx-5MS column. The mass detector ionization employed an electron impact of 70 eV. Compounds in the chromatograms were identified by comparison of their mass spectra with those in the NIST11 library database [42]. Chromatographic peaks were considered “unknown” when their similarity index (MATCH) and reverse similarity index (RMATCH) were less than 850 and discarded in this identification process [43]. These parameters referred to the degree in which the target spectrum matches the standard spectrum in the NIST Library (the value 1000 indicates a perfect fit), by comparison of their retention index with those reported in the literature [44], for the same type of column or those of commercial standards, when available. The retention indices were determined under the same operating conditions in relation to a homologous n-alkanes series (C8–C36) by

(1) $$\begin{array}{c} \text{RI}=100\times \left(\;\frac{n+Tr\left(unknown\right)-Tr\left(n\right)}{Tr\left(N\right)-Tr\left(n\right)}\;\right), \end{array}$$

where *n* is the number of carbon atoms in the smaller n-alkane, *N* is the number of carbon atoms in the larger n-alkane, and Tr is the retention time. Components relative concentrations were obtained by peak area normalization.

### 3.5. Fatty Acids Determination

The gas chromatography analysis was performed on an Agilent Technologies 7890B gas chromatograph using a 30 m capillary column, Supelco Omega-Wax, 0.25 mm (Agilent Corp. CA). Hydrogen was used as a carrier gas at a flow rate of 1 mL/min. Detection was with flame-ionization detection and areal quantitation was made with an auxiliary automatic integrator. A temperature of 170°C was used at the beginning, which was maintained for two minutes, rising from 2,5°C/min to a final temperature of 240°C, which was maintained for 3 min. Injector and detector remained at 250°C and 270°C, respectively. Methyl esters were prepared according to our previously described procedure [45] by treating the extracted oil with 0.5 M NaOH in methanol, followed by 14% BF3 in methanol treatment. The acid identification was made by comparing the relative retention times of FAME peaks from samples with standards. The results were recorded, processed, and expressed in relative percentage of each fatty acid.

### 3.6. Bacterial Strains

The uropathogenic bacteria were clinical isolates belonging to the Chemistry Department, Biological Tests Laboratory (Universidad Técnica Federico Santa María) collection. They comprised* Escherichia coli*,* Staphylococcus aureus*,* Klebsiella pneumoniae*, and* Proteus mirabilis*. The identification of all isolates was confirmed through genomic DNA analysis by sequencing (Macrogen USA, Cambridge, MA). Strains were cultured in Mueller-Hinton Broth (MHB) and Mueller-Hinton agar at 37°C.

### 3.7. *In Vitro* Antimicrobial Assay

The minimum inhibitory concentration (MIC) of the plant extract was determined using the broth serial dilution method with modifications, following Andrews 2001 [46]. Briefly, the extracts were first dissolved in ethanol (ET) and the final concentration of ET in each microwell was less than 1%, which did not affect the growth of the test strain. A dilution range of the extracts from 12.5 *μ*g/mL to 1600 *μ*g/mL was tested. Later, 1.5 *μ*L of microbial suspension (1 × 10^6^ CFU/mL) of each strain was inoculated into sterile 96-well microplates with equal volume of MHB broth and plant extracts. Then, the 96-well microplates were incubated aerobically at 37°C for 24 h in a shaker at 120 rpm. MIC was determined according to the OD_600_ obtained in an Accu Reader M965 spectrophotometer. After the cultures were incubated at 37°C for 24 h, the minimum inhibitory concentration (MIC) was confirmed as the lowest concentration of the test extract that demonstrated no visible growth. The plates were incubated for 24 h at 37°C. Chloramphenicol and streptomycin were used as positive control tested at the same concentrations for bacterial strains and 1% ET was used as negative control with inoculum. Also, a negative control was used with 1% ET without inoculum to subtract the OD_600_ obtained. Each concentration of the extracts was tested in triplicate. Each value represents the mean ± SD of three experiments performed in triplicate, for which the mean and standard deviation for each value were calculated.

Finally, the percentage of bacterial growth inhibition (GI) was calculated using the following formula:

(2) $$\begin{array}{c} GI\%=\frac{\left[\left(C_{Abs}-T_{Abs}\right)\right]}{C_{Abs}}*100, \end{array}$$

where *C*_Abs_ is the absorbance of the control treatment and *T*_Abs_ is the absorbance of samples treated with different extracts.

## 4. Conclusions

Different secondary metabolites of the n-hexane and ethyl acetate extracts were isolated from* T. officinale* (Compositae) leaves, mainly known as triterpenoids, and other unknown compounds to a lesser extent. Their presence and structures were elucidated by spectroscopic analyses. In addition, the fatty acids content was determined showing a higher content of palmitic and linolenic acids. Both extracts present antibacterial activity against uropathogenic clinical bacteria, being more active against Gram-positive bacteria (MIC 200 *μ*g/mL, 89% of bacterial inhibition). These results suggest that both extracts have potential as antibacterial uropathogenic disease agents.

## Figures and Tables

**Table 1:** Main components of Hex extract.

No.	RT (min)	Main components	RI^a^	% area^b^
1	14.74	2-Cyclohexen-1-ol	892	1.81
2	16.47	2-Cyclohexen-1-one	942	0.74
3	29.8	*α*-Ionene	1372	0.29
4	34.01	Brassicasterol acetate	1538	0.25
5	35.61	Diethyl phthalate	1605	4.25
6	40.77	3,7,11,15-Tetramethyl-2-hexadecen-1-ol	1841	13.08
7	40.91	4-Octadecenal	1848	0.39
8	43.15	n-Hexadecanoic acid	1960	1.05
9	43.82	Hexadecanoic acid, ethyl ester	1994	0.41
10	46.13	Phytol	2118	3.48
11	47.13	Ethyl 9,12,15-octadecatrienoate	2174	1.49
12	71.44	Lupeol	3443	23.31
13	75.13	Lupeol acetate	3516	25.09
14	75.26	Betulin	3518	23.81

^a^RI: retention indices relative to C_8_–C_36_ n-alkanes on the Rtx-5MS capillary column. ^b^Surface area of GC peak.

**Table 2:** Main components of AcOEt extract.

No.	RT (min)	Main components	RI^a^	% area^b^
1	13.64	1,2-Epoxycyclohexane	865	0.19
2	14.72	2-Cyclohexen-1-ol	892	0.90
3	16.44	2-Cyclohexen-1-one	941	0.41
4	40.68	Hexahydrofarnesol	1837	0.76
5	40.79	3,7,11,15-Tetramethyl-2-hexadecen-1-ol	1842	15.57
6	43.19	n-hexadecanoic acid	1962	0.83
7	43.83	Hexadecanoic acid, ethyl ester	1994	0.37
8	46.15	Phytol	2119	0.75
9	46.65	Linolenic acid	2147	0.67
10	47.01	Linoleic acid ethyl ester	2167	0.23
11	47.15	Ethyl 9,12,15-octadecatrienoate	2175	0.94
12	47.98	2-[(Z)-9-octadecenyloxyethanol]	2222	0.48
13	51.17	3-Ethyl-3-hydroxy-5*α*-androstan-17-one	2413	1.61
14	70.82	(22Z)-Stigmasta-5,22-diene-3*β*-ol acetate	3426	2.98
15	72.95	*β*-sitosterol	3483	4.55
16	73.43	*α*-Amyrin	3496	4.78
17	75.19	Lupeol acetate	3517	19.95
18	79.09	Betulin	3557	6.17

^a^RI: retention indices relative to C_8_–C_36_ n-alkanes on the Rtx-5MS capillary column. ^b^Surface area of GC peak.

**Table 3:** Fatty acids content in the *T. officinale* leaves collected from Temuco region, Chile (mg/g dry weight).

Fatty acid	mg/g dry weight of *T. officinale*, Temuco, Chile
C14 : 0 (myristic)	0,1
C16 : 0 (palmitic)	1,5
C18 : 0 (stearic)	0,3
C18 : 1 n-9 (oleic)	0,2
C18 : 2 n-6 (linoleic)	0,7
C18 : 3 n-3 (linolenic)	1,0
C20 : 0 (arachidonic)	0,1

**Table 4:** Antibacterial activity of n-hexane and ethyl acetate extract of *Taraxacum officinale* against the tested bacteria.

Extracts	Percentage of growth inhibition (%)/MIC (*µ*g/mL) at 24 h^*∗*^			
	*E. coli*	*S. aureus*	*K. pneumoniae*	*P. mirabilis*
Hex	72 ± 2.1 400	89 ± 3.3 200	52 ± 0.0 400	70 ± 0.8 800
EtOAc	97 ± 0.9 1600	0 ± 0.0	-	-
Chloramphenicol	95 ± 0.0 25	-	94 ± 0.0 100	89 ± 0.0 200
Streptomycin	-	88 ± 1.1 25	-	-

^*∗*^Mean of triplicates ± standard deviation of three replicates; (-) not tested.
